# Clinical sequencing: is WGS the better WES?

**DOI:** 10.1007/s00439-015-1631-9

**Published:** 2016-01-07

**Authors:** Janine Meienberg, Rémy Bruggmann, Konrad Oexle, Gabor Matyas

**Affiliations:** Center for Cardiovascular Genetics and Gene Diagnostics, Foundation for People with Rare Diseases, 8952 Schlieren-Zurich, Switzerland; Interfaculty Bioinformatics Unit and Swiss Institute of Bioinformatics, University of Berne, 3012 Berne, Switzerland; Zurich Center for Integrative Human Physiology, University of Zurich, 8057 Zurich, Switzerland

## Abstract

Current clinical next-generation sequencing is done by using gene panels and exome analysis, both of which involve selective capturing of target regions. However, capturing has limitations in sufficiently covering coding exons, especially GC-rich regions. We compared whole exome sequencing (WES) with the most recent PCR-free whole genome sequencing (WGS), showing that only the latter is able to provide hitherto unprecedented complete coverage of the coding region of the genome. Thus, from a clinical/technical point of view, WGS is the better WES so that capturing is no longer necessary for the most comprehensive genomic testing of Mendelian disorders.

**Electronic supplementary material:**

The online version of this article (doi:10.1007/s00439-015-1631-9) contains supplementary material, which is available to authorized users.

There is considerable discussion about the optimal application of next-generation sequencing (NGS) in the diagnosis of Mendelian disorders. Gene panels have been favored because of low sequencing costs, short turnaround time, and low rate of unspecific or incidental findings, while only about 10 % of the mutations detectable by whole exome sequencing (WES) were missed (Saudi Mendeliome Group 2015). In fact, gene panels related to the patients’ phenotype can be viewed as an inexpensive and rapid first-tier test. If this test is negative, WES or whole genome sequencing (WGS) can be considered as the most comprehensive second-tier test.

In WGS, genome-wide read coverage may allow reliable detection of copy number variations (CNVs), which can contribute substantially to disease burden (Girirajan et al. 2011). The prices of WGS are tumbling, turnaround time including data analysis (e.g., using GENALICE MAP, www.genalice.com) can be reduced to few days, virtual gene panels can be selected in silico to avoid incidental findings, and diagnostic yield may be as high as 73 %, surmounting conventional phenotype-directed single-gene analyses by up to one order of magnitude (Soden et al. 2014; Miller et al. 2015; Willig et al. 2015). Thus, WGS has to be considered as an alternative to WES.

We recently showed that even current WES platforms have problems in sufficiently capturing the whole exome and suggested that WGS, which forgoes capturing, is less sensitive to GC content and more likely than WES to provide complete coverage of the entire coding region of the genome (Meienberg et al. 2015). Here, we provide new insights into WGS, showing that the recently introduced PCR-free WGS offers hitherto unprecedented complete coverage of the coding region of the genome and, hence, that WGS instead of WES should be considered as the most comprehensive second-tier test.

We compared optimal WES (using Agilent SureSelect v5 + UTR capturing; Meienberg et al. 2015) with WGS (using Illumina’s TruSeq PCR-free WGS library preparation) in DNA samples of five females each. Sequencing was performed by vendors V2 (WES) and V4 (WGS) on a HiSeq 2000 at 100× and a HiSeq X Ten system at 60×, respectively. To largely reduce systematic errors and alignment artifacts, we restricted our comparison to RefSeq coding sequences which were uniquely mappable to X-chromosomal or autosomal regions (Derrien et al. 2012), identical in hg19 and hg38 genome assemblies, and not overlapping with common CNVs listed in the Database of Genomic Variants (DGV, MacDonald et al. 2014). For further details see electronic supplementary material.

Our current data show that novel PCR-free WGS is much less sensitive to GC content and leads to a more uniform coverage than WES and non-PCR-free WGS (Fig. 1a, Supplementary Figs. S1-S3). Although the average depth of coverage was less than half (65× in WGS versus 154× in WES, Supplementary Table S1), the number of RefSeq coding exons with complete (100 %) coverage at ≥13× was considerably larger in PCR-free WGS than in WES (100.00 vs. 98.15 %; Fig. 1b). The difference was more pronounced when the GC-rich first exons (59 vs. 51 % GC in all exons) were examined (100.00 % in PCR-free WGS vs. 93.60 % in WES; Fig. 1b). In the case of genes recommended by the American College of Medical Genetics (ACMG) to be reported if mutated (Green et al. 2013), PCR-free WGS completely covered all uniquely mappable exons (100 % at ≥13×) in all five samples of our study, whereas only 98.25 % of the ACMG exons were completely covered by WES, leading to complete WES coverage of only 75.56 % of the ACMG genes (Fig. 1b). A noticeable and clinically relevant difference in the performances of WES and WGS was also observed in the coverage of exons in which disease-causing mutations (DMs, including single nucleotide variants as well as small (≤20 bp) insertions, deletions, and indels) have been reported in HGMD (98.22 % in WES vs. 100.00 % in WGS; Fig. 1b). Accordingly, WES may fail to detect 0.42 % (401/95,118) of the currently known exonic DMs detectable by WGS. Considering the identification of non-coding pathogenic variation as well (Spielmann and Klopocki 2013), WES may miss a total of 0.81 % (863/106,819) of the DMs currently listed in HGMD and potentially detectable by WGS (99.19 % in WES vs. all but one DM in WGS; Fig. 1b). Notably, the 13× cutoff presented here reveals the minimum number of reads at which WGS achieves 100.00 % coverage in our samples. For the same WGS performance at the widely used 20× cutoff, sequencing at >100× ($$ 65*20$$/13) is needed (while for WES more sequencing reads may not result in more complete coverage due to capture limitations, especially in GC-rich regions).

**Fig. 1:**
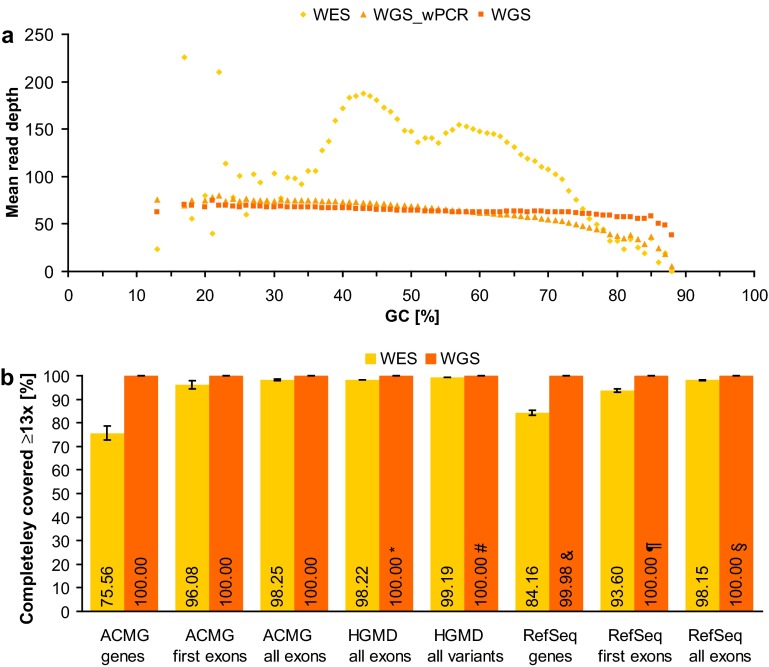
Performance comparison of WES and WGS. **a** Mean read depth of RefSeq coding exons per GC content shown for WES as well as for WGS with (WGS_wPCR) and without (WGS) PCR as means of five samples each. **b** Percentage of completely covered (i.e. ≥13 reads at each nucleotide position) genes, exons, and variants in WES and WGS without PCR as means of five samples each (error bars indicate 95 % confidence intervals). In the case of genes recommended for reporting by the ACMG (ACMG genes, *n* = 54) and of genes of the RefSeq database (RefSeq genes, *n* = 16,896), the set of all coding exons (ACMG all exons, *n* = 1152; RefSeq all exons, *n* = 177,084) and the set of start-codon-containing exons (first exons) were examined. The set of RefSeq exons harboring at least one disease-causing mutation (DM) listed in HGMD (HGMD all exons, *n* = 22,303) and the set of all coding and non-coding DMs (HGMD all variants, *n* = 106,819) were also analyzed. Note that 100.00 % implies a deviation of at most 0.005 %: *two exons were partially covered with 12 reads; ^#^one intronic mutation was covered with 12 reads; ^&^12 genes were partially covered with 7–12 reads; ^¶^three exons were partially covered with 10–12 reads; ^§^12 exons were partially covered with 7–12 reads

Furthermore, genome-wide uniformity of coverage makes WGS, rather than WES, suitable for CNV detection (Gilissen et al. 2014; Meienberg et al. 2015). In our samples, the coefficient of variation (cv = SD/mean) in coverage among the exons of an individual is on average about 4 times larger in WES than in PCR-free WGS (0.59 vs. 0.14). Admittedly, the relative lack of uniform coverage in WES does not appear to result from an increased noise level, since the inter-individual cv per exon is comparable in WES and WGS (0.08 vs. 0.09). In other words, the additional variability of WES coverage appears to be reproducible and, hence, can in principle be normalized in silico. However, such normalization algorithms are relatively complex, need to be calibrated for each enrichment protocol (Szatkiewicz et al. 2013), and allow only the detection of CNVs affecting the enriched genomic region. Moreover, gapless WGS also offers the detection of structural variants (SVs) based on paired and split reads, enabling the detection of (copy neutral) SVs at base-pair resolution (Escaramis et al. 2015). Thus, in our opinion, WGS will likely replace array techniques in CNV detection whereas WES might not.

WGS is available worldwide in laboratories that have high-throughput sequencing capacities of at least 60× $3*{10^9}$ bp as well as appropriate hard- and software resources to handle and interpret large WGS files. One may argue that WGS is more expensive than NGS with selective capturing of targets. Indeed, genetic mosaics and somatic cancer gene panels require several 100-fold sequencing depths to detect low-frequency non-reference variants, so that WGS would currently be too expensive for these applications. Otherwise, however, sequencing costs decline steadily and data interpretation efforts can be curtailed by in silico selection of relevant WGS parts. Considering that these parts are subject to change, selective capturing will require re-sequencing of unsolved cases, while with WGS only the re-analysis of existing data will be necessary. In addition, one may argue that WGS implies incidental findings of mutations not related to the patient’s present disease and findings of variants with uncertain or incomplete effect. Again, overload with such findings can be prevented by reducing the WGS data to virtual gene panels of interest. Thus, we and others (Belkadi et al. 2015; Lelieveld et al. 2015) believe that WGS is more powerful than WES in detecting exome variants so that future NGS diagnostics of Mendelian disorders will not involve capturing techniques anymore. In addition to previous studies, our present data show that PCR-free WGS provides an even more uniform and complete coverage of the exome than WGS with PCR during library preparation.

In conclusion, the performance of WES is sensitive to sequence (GC) content as well as capturing design and enrichment. Hence, WES does not entirely serve its purpose, whereas novel PCR-free WGS provides hitherto unprecedented complete coverage of the exome and other clinically relevant genomic sequences. The advantage of WGS therefore does not only include the identification of non-coding pathogenic variation, but, in view of its more complete exomic coverage as presented here, it is simply the better WES. As such, PCR-free WGS has to be considered as the most comprehensive second-tier genomic test. With sequencing costs further declining and by using appropriate virtual panels, WGS even has the potential to entirely replace WES and other techniques that involve selective capturing of target sequences.

## Electronic supplementary material

Supplementary material 1 (PDF 100 kb)
