# Supplementary material for: Clinical sequencing: is WGS the better WES?
Source: Hum Genet. 2016 Jan 7;135:359–62. doi: 10.1007/s00439-015-1631-9 (PMC4757617; doi:10.1007/s00439-015-1631-9)
Supplement: Supplementary file 1 — Supplementary material 1 (PDF 100 kb) [file 439_2015_1631_MOESM1_ESM.pdf]

Human Genetics, Short Report, Supplementary Information

Clinical Sequencing: Is WGS the better WES?

Janine Meienberg[1], Rémy Bruggmann[2], Konrad Oexle[1], Gabor Matyas[1,3]

[1] Center for Cardiovascular Genetics and Gene Diagnostics, Foundation for People with Rare Diseases, Schlieren-Zurich, CH-8952, Switzerland;

[2] Interfaculty Bioinformatics Unit and Swiss Institute of Bioinformatics, University of Berne, Berne, CH-3012, Switzerland;

[3] Zurich Center for Integrative Human Physiology, University of Zurich, Zurich, CH-8057, Switzerland;

Correspondence: matyas@genetikzentrum.ch

Materials and Methods

We compared optimal WES (using SureSelect v5+UTR capturing, Agilent Technologies, Santa Clara, CA, USA, and 3 µg input DNA; Meienberg et al. 2015) with WGS (using TruSeq PCR-free WGS library preparation, Illumina, San Diego, CA, USA, and 2-3 µg input DNA) in DNA samples of five females each. Sequencing was performed by vendors V2 (WES) and V4 (WGS) on a HiSeq 2000 (Illumina) at 100× and a HiSeq X Ten system (Illumina) at 60×, respectively, as previously described (Meienberg et al. 2015). For the analysis of coverage in dependence of GC content, we also considered WGS with PCR during library preparation of five females sequenced at 60× on a HiSeq X Ten sequencer (Illumina) by V4 using the TruSeq Nano DNA Sample Preparation Kit (Illumina) for 350-bp insert size and 2-3 µg input DNA (WGS wPCR, Figure 1a, Supplementary Figures S1-S3). To largely reduce systematic errors and alignment artifacts, we restricted our comparison to RefSeq coding sequences which were uniquely mappable (i.e., 75-bp mappability = 1, http://genome.ucsc.edu, wgEncodeCrgMapabilityAlign75mer, Derrien et al. 2012) to X-chromosomal or autosomal regions, identical in hg19 and hg38 genome assemblies, and not overlapping with common CNVs detected in more than 90 individuals according to the Database of Genomic Variants (DGV, July 2015, http://genome.ucsc.edu, MacDonald et al. 2014). Disease-causing mutations (DMs) of the Human Gene Mutation Database (HGMD Professional 2015.2, http://www.biobase-international.com) located within RefSeq coding exons fulfilling these filter criteria were included, whereas for non-coding DMs unique mappability of 75-bp flanking sequences, absence of overlap with common CNVs listed in DGV and identity to hg38 of 150-bp flanking sequences were used for filtering. First exons are defined as the coding part of the exon containing the start codon. The number of reads and the coverage at defined minimal read depth as well as the GC content based on at least 60 bp (i.e., the appropriate number of flanking sequences were included for smaller exons) were determined according to our previous study (Meienberg et al. 2015).

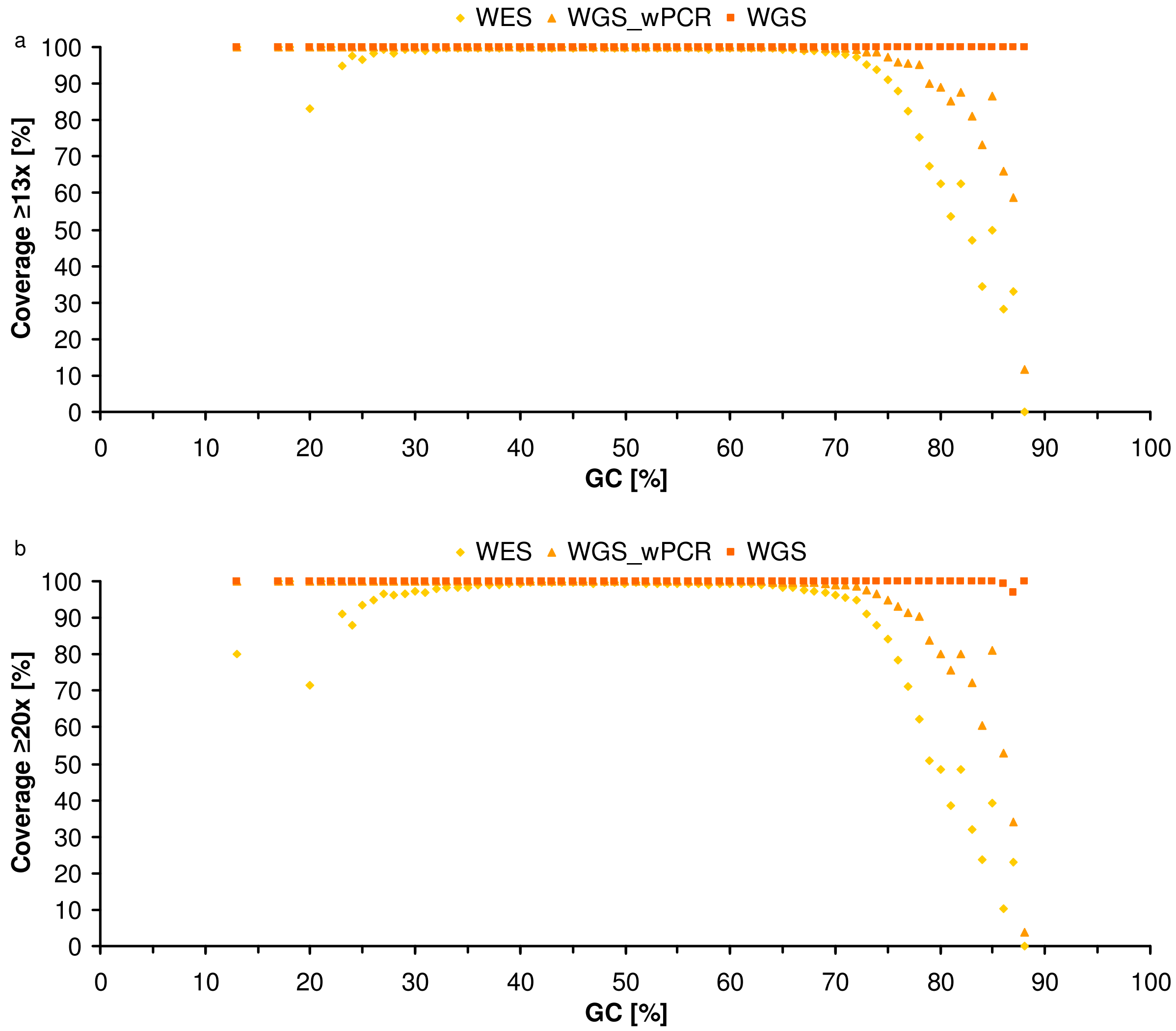

Supplementary Fig. S1 Mean coverage of RefSeq coding exons fulfilling the filter criteria per GC content shown for WES as well as for WGS with (WGS_wPCR) and without (WGS) PCR as means of five samples each. (**a**) Mean coverage at ≥13 reads. (**b**) Mean coverage at ≥20 reads

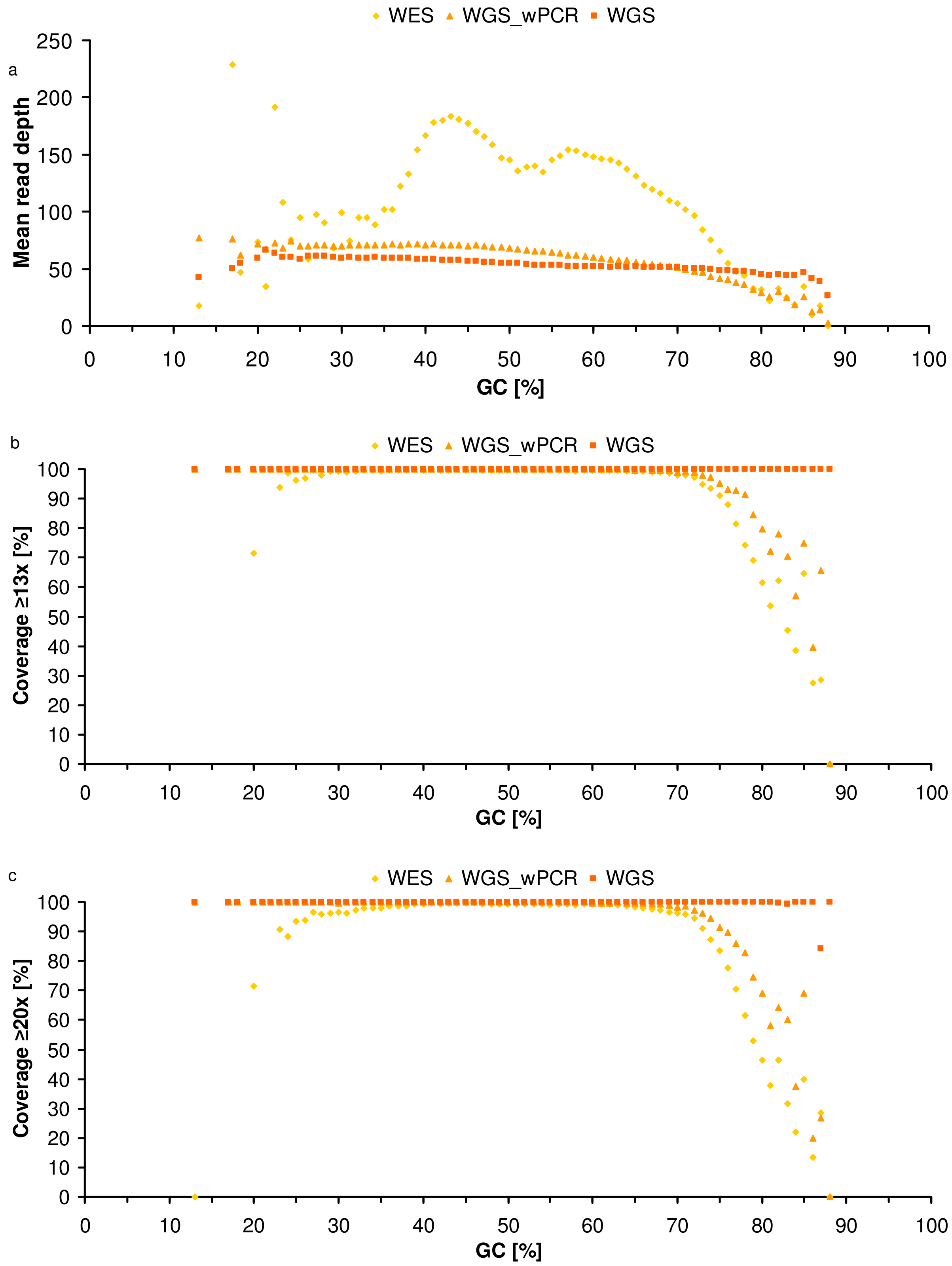

Supplementary Fig. S2 Mean read depth and coverage of RefSeq coding exons fulfilling the filter criteria per GC content for the same DNA sample analyzed by all three NGS methods. (**a**) Mean read depth. (**b**) Mean coverage at ≥13 reads. (**c**) Mean coverage at ≥20 reads. Data were determined for WES as well as for WGS with (WGS_wPCR) and without (WGS) PCR during library preparation

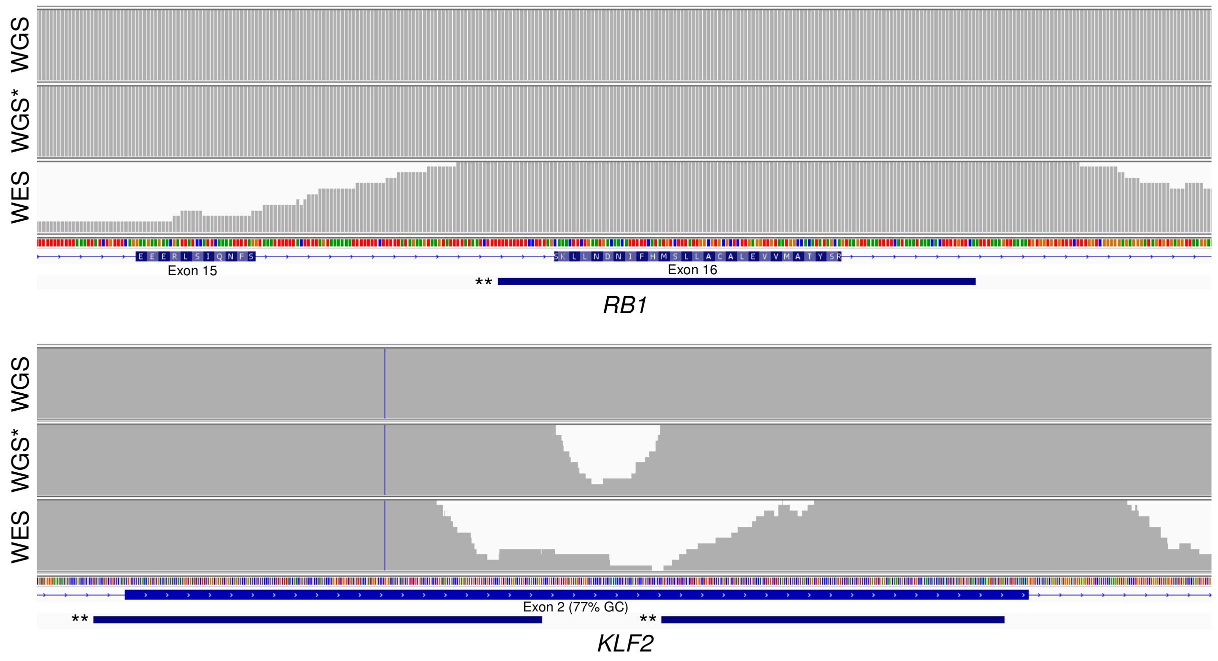

Supplementary Fig. S3 Difference in coverage between WES and WGS for two genes. In case of *RB1* (top) not all exons are captured so that WES is incomplete as opposed to WGS. In case of *KLF2*, GC-rich (77%) exon 2 is completely covered by PCR-free WGS and almost completely by WGS with PCR while a large gap in coverage is present in WES. Coverage tracks are visualized by the Integrative Genomics Viewer (IGV, https://www.broadinstitute.org/igv) and display ranges are set to 0-13 reads. *, WGS with PCR during library preparation; **,designed target region of Agilent SureSelect v5+UTR

Supplementary Table S1 Run statistics

WES				WGS			
**Sample**	**Raw reads**	**Aligned reads (%)[a]**	**Exome coverage[b]**	**Sample**	**Raw reads**	**Aligned reads (%)[a]**	**Exome coverage[b]**
**44**	188'901'528	172'118'729 (91.1%)	168×	**3772**	1'582'096'450	1'342'203'113 (84.8%)	67×
**280**	173'953'326	160'900'330 (92.5%)	154×	**8034**	1'722'656'754	1'452'000'679 (84.3%)	73×
**326**	173'729'966	157'585'695 (90.7%)	151×	**8036**	1'588'114'742	1'315'026'807 (82.8%)	67×
**7344**	172'157'056	156'320'331 (90.8%)	147×	**8217**	1'567'669'540	1'219'483'563 (77.8%)	61×
**7739[c]**	178'561'090	156'695'689 (87.8%)	153×	**7739[c]**	1'305'531'238	1'116'476'500 (85.5%)	55×
**Mean**	**177'460'593**	**160'724'155 (90.6%)**	**154×**	**Mean**	**1'553'213'745**	**1'289'038'132 (83.0%)**	**65×**

[a]Total mapped and deduplicated read counts (% of raw reads)
[b]Calculated as total number of aligned bases of the exome divided by the length of the exome, thereby considering only RefSeq coding exons fulfilling our filter criteria (see Supplementary Materials and Methods)
[c]Same sample sequenced with both approaches
